# Redefining serological diagnostics with immunoaffinity proteomics

**DOI:** 10.1186/s12014-023-09431-y

**Published:** 2023-10-12

**Authors:** Jonathan Walter, Zicki Eludin, Andrei P. Drabovich

**Affiliations:** Division of Analytical and Environmental Toxicology, Department of Laboratory Medicine and Pathology, Faculty of Medicine and Dentistry, University of Alberta, 10-102 Clinical Sciences Building, Edmonton, AB T6G 2G3 Canada

**Keywords:** Serological diagnostics, Immunoglobulins, Proteomics, Mass spectrometry, Immunoassays.

## Abstract

Serological diagnostics is generally defined as the detection of specific human immunoglobulins developed against viral, bacterial, or parasitic diseases. Serological tests facilitate the detection of past infections, evaluate immune status, and provide prognostic information. Serological assays were traditionally implemented as indirect immunoassays, and their design has not changed for decades. The advantages of straightforward setup and manufacturing, analytical sensitivity and specificity, affordability, and high-throughput measurements were accompanied by limitations such as semi-quantitative measurements, lack of universal reference standards, potential cross-reactivity, and challenges with multiplexing the complete panel of human immunoglobulin isotypes and subclasses. Redesign of conventional serological tests to include multiplex quantification of immunoglobulin isotypes and subclasses, utilize universal reference standards, and minimize cross-reactivity and non-specific binding will facilitate the development of assays with higher diagnostic specificity. Improved serological assays with higher diagnostic specificity will enable screenings of asymptomatic populations and may provide earlier detection of infectious diseases, autoimmune disorders, and cancer. In this review, we present the major clinical needs for serological diagnostics, overview conventional immunoassay detection techniques, present the emerging immunoassay detection technologies, and discuss in detail the advantages and limitations of mass spectrometry and immunoaffinity proteomics for serological diagnostics. Finally, we explore the design of novel immunoaffinity-proteomic assays to evaluate cell-mediated immunity and advance the sequencing of clinically relevant immunoglobulins.

## Background

Serological diagnostics is generally defined as the detection of the specific immunoglobulins developed against viral, bacterial, or parasitic diseases and circulating in patient blood or proximal fluids. Serological tests present simple and informative tools for clinical diagnostics and facilitate the detection of previous and current infections, evaluation of immune status, and disease prognosis. Serological tests date back to 1906 and the development of the Wassermann reaction, a complement fixation test to detect anti-cardiolipin antibodies and diagnose syphilis [1]. Currently, serological tests provide complementary clinical information and allow for earlier and more accurate diagnosis of a variety of non-infectious diseases including autoimmune disorders, cancer, celiac disease, rheumatoid arthritis, and others [2–4].

The conventional serological tests are exclusively based on the concept of indirect Enzyme-Linked Immunosorbent Assays (ELISA) or their modifications, such as Lateral Flow Immunoassays (LFI) or Fluorophore Linked Immunosorbent Assays (FLISA). Indirect immunoassays, however, may suffer from non-specific binding and cross-reactivity, which result in higher false positive rates and lower diagnostic specificity. Lower diagnostic specificity prohibits the use of serological tests to screen populations with low disease prevalence (early stages of a pandemic, rare diseases, asymptomatic general populations, etc.) [5]. Furthermore, the complete panel of human immunoglobulin isotypes (IgG, IgM, IgA, IgE, and IgD) and subclasses (IgG1, IgG2, IgG3, IgG4, IgA1, and IgA2) is rarely evaluated; the majority of serological studies traditionally evaluate only the antigen-specific total IgG, total IgA, IgM, or their combinations [6]. Redesign of conventional serological tests to evaluate and minimize cross-reactivity, utilize universal reference standards, and measure disease-relevant subclasses and isotypes would facilitate the development of assays with higher diagnostic specificity, reliable implementation, and correct interpretation of serological tests for diagnosis of lower-prevalence diseases, screening of asymptomatic populations, and earlier detection [7–10].

Innovative and comprehensive immunoassay detection technologies such as mass spectrometry and protein microarrays promise to resolve limitations of conventional indirect immunoassays and provide multiplex measurements of immunoglobulin isotypes and subclasses, allowing for a more detailed analysis of disease states and improving disease prognosis [10]. In this review, we discuss the current clinical needs for serological diagnostics, overview the conventional and emerging immunoassay detection technologies, discuss in detail proteomics and mass spectrometry as a comprehensive immunoassay detection technology, and explore the design of novel immunoaffinity-proteomic assays to evaluate cell-mediated immunity and advance sequencing of clinically-relevant immunoglobulins.

## Main text

### Clinical needs addressed with serological diagnostics

#### Diagnostics of infectious diseases

Serological testing remains an essential tool to aid PCR and RT-PCR diagnostics of infectious diseases and provide complimentary clinical information (Table 1). For example, prenatal screening for infectious diseases is aimed at preventing the transfer of infections from mother to child before birth thus identifying and decreasing the risk of pregnancy complications [11]. Around 40% of pregnancies with syphilis infections result in the death of the fetus while surviving newborns could develop a wide array of physiological abnormalities [11]. Likewise, hepatitis B (HBV) infections can lead to serious complications, and 90% of infected infants develop chronic HBV infections [12]. Serological testing, such as the detection of anti-HBc IgM, can assist in the prognosis of infections, allowing for the appropriate preventative measures [12].

**Table 1:** Clinical needs addressed with serological testing

Clinical Need	Disease/Pathogen/Agent	Isotypes (measured routinely)	Major subclasses (not measured routinely)	References
*Prenatal screening*	Rubella	IgG, IgM	IgG1, IgG3	[181]
	Varicella	IgG, IgM	IgG1, IgG3	[182]
	HIV	IgG, IgM	IgG1, IgG3	[183]
	Syphilis	IgG, IgM	IgG1, IgG3	[184]
	Chlamydia	IgG, IgM	IgG1, IgG3	[185]
*Blood transfusion*	Babesiosis	IgG, IgM, IgA		[186]
	Malaria	IgG, IgM, IgA		[187]
*Viral hepatitis*	Hepatitis A, B, C, D, E	IgG, IgM	IgG1, IgG3	[14, 16]
*Streptococcal Infections*	Streptococcus pneumoniae	IgG, IgM	*IgG2*	[188]
*Travel-related Infections*	Yellow Fever	IgG, IgM		[19]
	Typhoid Fever	IgG		[20]
	Influenza Virus	IgG	IgG1, IgG3	[22]
*Emerging infections*	COVID-19	IgG, IgM, IgA	IgG1, IgG3	[10, 28]
	Dengue	IgG, IgM		[24]
	West Nile Virus	IgG, IgM		[25]
*Autoimmune diseases*	Rheumatoid arthritis	IgG, IgM	IgG1, *IgG4*	[2, 189]
	Crohn’s disease	IgA, IgG	IgG1, *IgG2*	[33, 190]
	Ulcerative colitis	IgA, IgG	IgG1, *IgG2*	[34, 190]
	Autoimmune gastritis	IgG, IgM		[191]
	Celiac disease	IgA, IgG	IgG1, IgG3	[3]
	Multiple sclerosis	IgG, IgM	IgG1, *IgG2*	[41]
	Systemic lupus erythematosus	IgA, IgG, IgM	IgG1, *IgG4*	[44, 45]
	IgG4-related diseases	IgG	*IgG4*	[192]
*Amyloidosis*	Amyloid fibrils	IgA, IgG, IgM	IgG1	[193]
*Cancer*	General	IgA, IgE, IgG, IgM	IgG1	[8, 55]
	Cancer/testis antigens	IgG		[194]
	Mutated proteins	IgA, IgG, IgM		[195, 196]
	Gene fusion proteins	unknown; CD4^+^ and CD8^+^ T lymphocytes		[197, 198]
*Infertility*	Spermatozoa proteins	IgA, IgG, IgM	IgG1, IgG3	[51]
*Allergy*	Numerous allergens	IgE, IgG	*IgG4*	[199]

Blood transfusion is another area where serological testing for infectious diseases is critical. Blood transfusions have revolutionized hematologic treatments, and there have been increases in safety measures over the last few decades to eliminate infections transmitted during transfusion [13]. Transfusion complications are a relatively common phenomenon and remain a serious concern. Notably, arboviruses, bacteria, and parasites present the most common sources of transfusion-transmitted infections [13]. For example, malaria infections through blood transfusion remain a concern in malaria-endemic and non-endemic countries due to travel and lack of screening [13]. Babesiosis, a zoonotic disease caused by tick-borne piroplasmids, presents another risk to the recipient population [13].

Viral hepatitis and the associated inflammation of the liver is one of the leading causes of mortality worldwide [14]. Five different hepatitis viruses A to E, each of a distinct viral family and with numerous genotypes, lead to viral hepatitis [14]. Hepatitis A (HAV), B (HBV), and C (HCV) viruses include 7, 10, and 7 genotypes, respectively [12, 14, 15]. Hepatitis D virus (HDV) has 8 genotypes and relies on coinfection with HBV and its lipid envelope (HBsAg) for replication [14]. Hepatitis E virus (HEV) has 4 genotypes but a single serotype [14, 16]. Upon hepatitis infection, specific IgM immunoglobulins are produced and manifest an acute infection [14, 16]. HBV testing is a prominent example of detailed serological diagnostics which evaluates the presence of viral protein antigens and the corresponding IgG and IgM; different combinations of positive and negative outcomes for protein antigens and antibodies provide a detailed interpretation of HBV status, such as acute, chronic, past infections, etc. [17]. Elimination of inconsistencies with diagnostics sensitivity and specificity of serological testing for hepatitis viruses and their distinct genotypes is a recognized need [16].

Travel-related infectious diseases represent another area where diagnosis and vaccination status rely on accurate and timely serological testing. Yellow fever, an arbovirus transmitted by mosquitos [18, 19], is currently one of the biggest concerns for travelers [18]. Yellow fever vaccinations provide long-term protection but are not required in non-endemic countries. Typhoid fever mediated by the gram-negative *Salmonella* bacteria also remains a large concern [20]. While vaccinations result in sustained serum IgG antibodies [20], typhoid fever serological tests require further validation to be used in clinics [21]. Influenza virus remains a concern due to its high mutation rates and adaptability, but its epidemics could be prevented through vaccinations and assessment of antibody protection in regions susceptible to the rapid evolution and adaptability of influenza [22].

Emerging infections in populations lacking prior immunity present global health concerns but also opportunities for the rapid implementation of innovative serological assays. Dengue fever, a re-emerging disease caused by the dengue virus, has 4 different serotypes of IgM and IgG [23]. Pre-existing antibodies often result in dengue hemorrhagic fever in patients with secondary dengue infection [24]. West Nile Virus, a mosquito-transmitted disease, triggers the IgM and IgG response [25] and raises concerns due to high morbidity rates [25].

The novel SARS-CoV-2 coronavirus and the COVID-19 pandemic have recently impacted the entire world. At the early stages of the COVID-19 pandemic novel serological tests were not thoroughly validated and reported diagnostic specificity as low as 95%, preventing the correct interpretation of test results [5]. To achieve positive predictive values > 90%, serological tests with 95% diagnostic specificity could only be informative in populations with COVID-19 prevalence > 33%. At the early stages of the pandemic (~ 0.1% prevalence estimated by RT-PCR), the poorly validated serological assays resulted in highly over-estimated and vastly incorrect rates of asymptomatic disease and “herd immunity” [26, 27], potentially undermining the public trust in the evidence-based medicine. The lessons learned demonstrated the importance of the rational design and development of serological tests and the need for thorough and independent validation of their diagnostic performance [10]. It should also be mentioned that the humoral immune response to SARS-CoV-2 has been thoroughly evaluated mostly for IgM, IgG, and IgA isotypes [28, 29], while the evaluation of the dynamics and cooperation of IgG1-4 and IgA1-2 subclasses could provide additional knowledge on the complexity of humoral and cellular immune responses [10].

#### Autoimmune diseases

Rheumatoid arthritis is the most common inflammatory autoimmune disease of the joints with a prevalence of 0.5-1% [30]. Serological testing for anti-rheumatoid factor IgM and anti-citrullinated protein IgG antibodies is useful to assist with the diagnosis and provide early interventions, to mitigate symptoms [2]. However, 30–50% of individuals with confirmed rheumatoid arthritis test negative [2]. Improved serological testing for a variety of antigens and distinct IgG subclasses could improve early diagnosis of asymptomatic rheumatoid arthritis [7].

The development of specific serological tests will greatly benefit the diagnosis and treatment of inflammatory bowel diseases, such as Crohn’s disease and ulcerative colitis, which significantly affect the quality of life, increase the risk of death, and are currently incurable [31, 32]. Multiple antibodies have been tested for diagnosis of the specific types of inflammatory bowel diseases, primarily anti-*Saccharomyces cerevisiae* IgG and IgA antibodies and anti-neutrophil cytoplasmic antibodies [33, 34]. The serological testing could distinguish between Crohn’s disease and ulcerative colitis but suffered from low diagnostic sensitivity [33, 34]. Detailed investigation of the levels of antibody isotypes and subclasses against a variety of antigens could aid in earlier and more specific diagnosis of inflammatory bowel diseases [35].

Autoimmune gastritis, an inflammatory autoimmune disorder, may lead to chronic atrophic gastritis followed by pernicious anemia [36, 37]. Autoimmune gastritis is typically asymptomatic until pernicious anemia develops and is then diagnosed by endoscopic biopsy [36]. Serological tests for antibodies targeting parietal cells and intrinsic Castle’s factor are informative for initial assessment but their diagnostic specificity and sensitivity are not sufficient to replace diagnostic biopsies [36, 37].

Celiac disease is another chronic autoimmune disorder characterized by inflammation of the small intestine in response to gluten [38]. It is associated with an increased risk of death and decreased quality of life due to a variety of complications including gastrointestinal distress, malabsorption, and anemia [38]. Diagnosis is based on the detection of anti-tissue transglutaminase and anti-endomysial IgA antibodies followed by an endoscopic duodenal biopsy. In some populations, such as symptomatic children, a diagnosis can be made based on serology alone [3]. However, nearly 10% of patients with confirmed celiac disease on biopsy tested negative for the established serological markers [39]. Testing the levels of specific antibody subclasses in celiac disease may facilitate more accurate diagnosis and potentially reduce the need for biopsies.

Multiple sclerosis is a major contributor to neurological-derived disability in young adults [40]. Diagnosis of multiple sclerosis is based on symptoms, imaging, and analysis of cerebrospinal fluid for IgG and IgM [41]. Early diagnosis and treatment help improve outcomes of multiple sclerosis [42].

Systemic lupus erythematosus (SLE), a chronic autoimmune disorder, could be manifested either through only minor symptoms, such as skin rashes, or a variety of severe symptoms such as organ failures [43]. SLE diagnosis is complex and is based on several criteria or biopsy confirmations [44]. The presence of antinuclear IgG or IgM autoantibodies targeting DNA, phospholipids, and nuclear antigens is included in the diagnostic criteria [44, 45].

#### Infertility

Globally, nearly 15% of couples are infertile, and around half of those cases are due to male factor infertility [46–50]. Antisperm antibodies (ASA), primarily of IgA and IgG isotypes, were detected in nearly 16% of infertile men and were associated with male immune infertility [51]. ASA were detected either directly, as bound to sperm cell surface, or indirectly, as soluble antibodies [51]. While the identity of the ASA antigens was not studied in detail, the cell-surface testis-specific proteins could be potential candidates [52]. It should be noted that about 2% of fertile men test positive for ASA, so the presence of ASA alone is not sufficient to diagnose immune infertility [51]. Analysis of data on ASA prevalence and their impact on fertility was complicated by the variability of tests, sample types, and thresholds used to report a positive result [51, 53].

#### Cancer

Detection of autoantibodies generated against specific tumor-associated antigens (TAAs), such as cancer/testis antigens, may facilitate early cancer diagnosis and prognosis [8, 9, 54]. Autoantibodies could potentially be detected in serum before cancer becomes clinically significant [8, 55]. Serological diagnostics of cancers, however, have not been well established. Common challenges of cancer autoantibody testing include insufficient understanding of the identity of cancer-specific antigens, high analytical sensitivity of assays to detect extremely low levels of antigens secreted by small tumors and transient levels of the corresponding autoantibodies, lack of tools for the independent evaluation of diagnostic specificity of serological tests, and lack of standards [9].

Over the past decades, autoantibodies were identified in serum of patients with breast, lung, ovarian, and prostate cancers [8, 55]. For example, CA125 in combination with the human epididymis protein 4 antigen-autoantibody complexes increased sensitivity from 63 to 81% to detect early-stage ovarian cancer [4]. Several autoantibody panels including Videssa Breast [56], EarlyCDT-Lung [57], and MitogenDx [58] are now available as Laboratory Developed Tests and justify the use of autoantibodies as cancer biomarkers. MitogenDx cancer test to diagnose the paraneoplastic syndrome, often the first manifestation of neoplasms [59], measures autoantibodies against several testis- and brain-specific proteins in lung, breast, ovarian, and other major cancers [60]. There was also a strong association found between paraneoplastic syndrome, IgG4-related disease, and cancer [61, 62]. Previous studies detected IgG, IgA, and IgE autoantibodies [8, 55, 63] but rarely evaluated autoantibody subclasses. Few studies revealed IgG1 and IgG3 as the most abundant cancer autoantibodies [64, 65]. Interestingly, IgG4, a “blocking” antibody subclass often generated after long-term exposure to antigens in non-infectious settings [66], was found associated with immune evasion, immunotherapy inefficiency, and poor survival in cancer [67–69]. Innovative high-specificity assays for serological testing may revolutionize cancer autoantibody studies, validate cancer/testis antigen hypothesis, discover novel immunotherapy targets, and enable precision approaches to immunotherapy.

### Serological diagnostics utilizing conventional immunoassay detection techniques

Since measurements of pathogen-specific antibodies circulating in serum rely on highly specific antigen-antibody interactions and affinity enrichments, serological assays could be generalized as immunoassays. The only exception of serological testing implemented without the requirement for antigen-antibody affinity interactions would be a detection of ‘M-proteins’ in monoclonal gammopathy or multiple myeloma [70]. ‘M-proteins’ can be directly measured by LC-MS or other techniques due to the extremely high levels in patient serum (up to 30 mg/mL) and monoclonal sequences [71, 72]. In this review, we discuss immunoassays with either conventional detection techniques, such as enzyme-linked absorbance measurements, or the emerging detection technologies, such as mass spectrometry. Other differences between serological assays include direct or indirect detection of antibody constant heavy chains, label-based or label-free approaches, multiplexing capabilities, affordability, analytical sensitivity and specificity, reproducibility, and throughput. In this section, we will overview immunoassays with conventional detection techniques.

#### Indirect ELISA

**Fig. 1:**
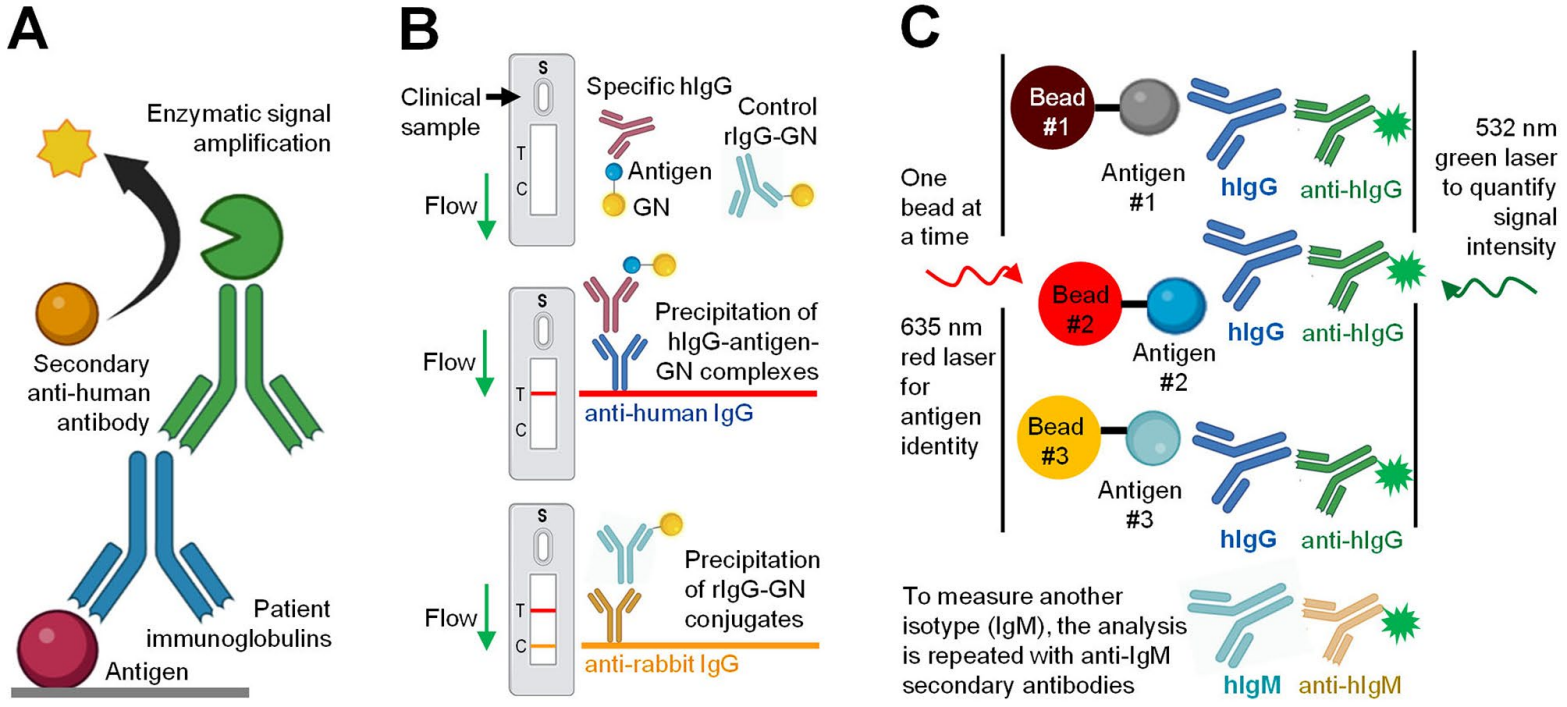
**Serological immunoassays with conventional detection techniques**. **(A) Colorimetric indirect ELISA**: antigens of interest are immobilized on the microplate surface and incubated with diluted blood serum samples. Specific human antibodies (blue) are captured, and non-specific antibodies and proteins are removed with microplate washing. Enzyme-conjugated secondary anti-human antibodies (green) oxidize the substrate (yellow), and the absorbance of the product is measured by a spectrophotometer. Relative antibody titers are determined by the highest dilution of a positive blood serum sample that provided a positive result. **(B) Lateral flow immunoassay**: Specific antibodies in patient samples bind to an antigen immobilized on colloidal gold nanoparticles on a sample pad (S). Capillary flow transfers complexes to the conjugation pad, where the complexes interact with the immobilized anti-human secondary antibodies, aggregate, and precipitate at the test line (T). Precipitation of gold nanoparticles results in color change (red stripe). As a control for test completion, rabbit antibodies conjugated to gold nanoparticles travel along through the T region, interact with the goat-anti-rabbit antibodies at the control line C, precipitate, and result in color change. **(C) Multiplex particle-based flow cytometry**: Each antigen is conjugated to a bead of a unique “color” which is predetermined by a unique combination of ten infrared dyes at different concentrations. Beads are mixed and incubated with serum, and the antigens capture corresponding human antibodies. Secondary anti-human IgG antibodies are conjugated to a fluorescent dye (green) used for quantification. Particle-based flow cytometry utilizes two different lasers to detect the bead identity (red laser) and signal intensity (green) of a single bead passing through the detection region. To map antibody isotypes and subclasses across multiple antigens, the analysis is repeated with the secondary antibodies specific for human IgM, IgA, or IgG1-4

Enzyme-linked immunosorbent assay (ELISA) is a well-established technique in research and clinical laboratories [6]. Indirect ELISA (Fig. 1A) relies on affinity enrichment of antigen-specific polyclonal antibodies followed by their detection using the secondary anti-human antibodies conjugated to an enzyme (horseradish peroxidase, alkaline phosphatase, or beta-galactosidase). In the case of horseradish peroxidase, the signal amplification is provided through the enzyme-catalyzed conversion of 3,3′,5,5′-tetramethylbenzidine substrate into its oxidized form which has a specific absorbance at 450 nm [73, 74]. In a fluorophore-linked immunosorbent assay (FLISA), secondary antibodies are conjugated to a fluorescent molecule. While indirect ELISA is a highly sensitive assay, it suffers from non-specific binding (such as non-specific adsorption of analytes and reagents to the microplate surface), cross-reactivity (such as cross-reactivity of secondary antibodies), and challenges with assay standardization [6]. Due to the lack of established standards of antigen-specific human polyclonal antibodies, indirect ELISA measurements typically report relative units, such as signal intensities, binding antibody units (BAU)/mL, or antibody ‘titers’ (the highest sample dilution factors which result in positive signals), but not absolute concentrations (µg/mL). Relative measurements are considered one of the major limitations which restrict inter-hospital and international standardization of serological testing [75]. The conventional immunoassays could hardly be multiplexed for the complete panel of human antibody isotypes and subclasses due to the cross-reactivity of the secondary antibodies, lack of the multiple spectrally-resolved fluorescent labels, and the limited dynamic range resulting in the need to measure multiple dilutions of the same sample [76, 77].

#### Lateral Flow Immunoassay

Lateral Flow Immunoassay (LFI; Fig. 1B) is a variant of indirect immunoassay intended for rapid, straightforward, instrument-free, and point-of-care detection of pathogen-specific antibodies in a variety of clinical samples [78]. A paper-based LFI device is composed of several compartments, including a sample pad, conjugation pad, test line (immobilized anti-human IgG antibody), control line (immobilized anti-rabbit IgG antibody), and an absorbent pad [78]. LFI limitations include those of indirect ELISA (cross-reactivity and challenges with standardization), as well as semi-quantitative measurements, lower sensitivity and reproducibility, and batch-to-batch variability [79].

#### Multiplexed particle-based flow cytometry

Particle-based flow cytometry immunoassays were developed to enable multiplexed measurements. The most common platforms, such as Luminex assays (Fig. 1C), comprised highly multiplexed bead-based immunoassays in which dozens of different analytes were measured in a single sample [80]. Particle-based flow cytometry immunoassays are robust, reproducible, require minimal expertise, and are widely used in clinical and research laboratories.

### Serological diagnostics utilizing the emerging immunoassay detection technologies

**Fig. 2:**
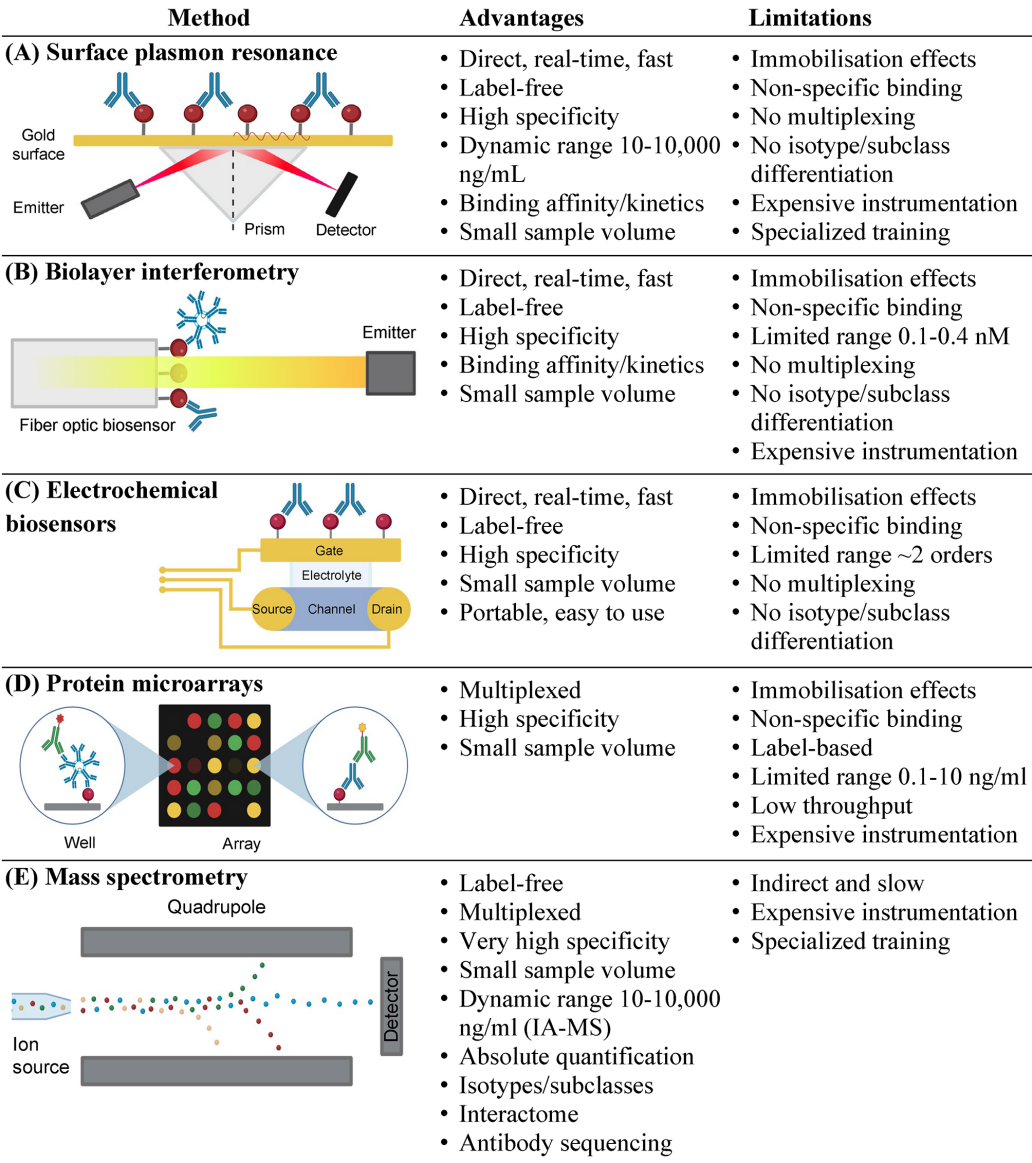
**Emerging immunoassay detection technologies for serological diagnostics**. **(A)** Surface plasmon resonance assays detect antibodies through their binding to antigens immobilized on gold surfaces. Specific binding results in plasmon resonance and changes in the refractive index. **(B)** A biolayer interferometry immunosorbent assay detects the shift in the wavelength of the reflected light upon antigen-specific antibody binding to the surface of a fiber optic sensor. **(C)** Electrochemical biosensors are transistors that detect antibody-antigen binding onto a gate electrode and measure changes in voltage across the source and drain electrodes. **(D)** Protein microarrays consist of numerous antigens that are printed onto the surface, enrich specific antibodies, and facilitate their relative quantification using fluorophore-labeled secondary antibodies. **(E)** Following affinity enrichment and trypsin digestion, MS facilitates highly specific quantification of peptides representing human immunoglobulin isotypes and subclasses

Recent infectious disease epidemics and pandemics have accelerated the development of innovative approaches for serological testing (Fig. 2).

#### Surface plasmon resonance (SPR) biosensors

Surface plasmon resonance (SPR) biosensors present an emerging approach for serological diagnostics with direct label-free quantification [81]. Its major limitations include relatively poor analytical sensitivity, low spatial resolution, expensive equipment, and extensive training of personnel [82, 83]. Rapid detection of protein biomarkers, human antibodies, and monoclonal antibody therapeutics is a promising application of SPR biosensors [84, 85].

#### Biolayer interferometry

Similar to surface plasmon resonance, biolayer interferometry (BLI) provides fast, label-free, and real-time detection of antigen-antibody interactions and provides information about interaction affinity and kinetics [86–88]. Some examples of BLI assays include and label-free measurements of monoclonal antibody therapeutics (LOQs 2–10 µg/mL in serum) and detection of anti-COVID-19 antibodies [89–92].

#### Electrochemical biosensors

Electrochemical biosensors are becoming an increasingly useful diagnostic tool and can rapidly detect proteins in biological fluids [93, 94]. Some recent examples include the detection of tau protein and its interactions [95] and measurements of COVID-19 mRNA, proteins, and serological antibodies [96].

#### Protein microarrays

Protein microarrays facilitate simultaneous analysis of thousands of human proteins and their functional interactions [97–99]. Ultra-high-density ‘human proteome’ microarrays (HuProt, ProtoArray, NAPPA, etc.) enable serological profiling of hundreds of patient samples across > 20,000 full-length human protein antigens [100–102]. Limitations of protein microarrays include challenges with standardization, high costs, and lack of clinical translation.

### Immunoaffinity – mass spectrometry for serological diagnostics

#### Overview of mass spectrometry technologies for proteomics

Mass spectrometry (MS) technologies, with their numerous approaches to measuring the variety of analytes ranging from small molecules to large proteins and intact viral particles, are rapidly reshaping clinical laboratories. Protein analysis is currently dominated by the bottom-up proteomic approaches, in which unique enzyme-derived peptides are used as proxies for the identification and quantification of the corresponding proteins [103, 104]. While trypsin is still the most common enzyme, alternative proteases (Lys-C, Glu-C, chymotrypsin, etc.) have been actively investigated to complement trypsin [105]. Common LC-MS proteomic workflows include LC separation of peptides, peptide ionization, separation of ions by their mass-over-charge (m/z) ratios, and measurements of the intensity of each molecular ion [106]. Ionization of thermally labile biological molecules, such as proteins, is typically achieved with soft ionization techniques including electrospray ionization (ESI) and matrix-assisted laser desorption/ionization (MALDI). A variety of mass analyzers are utilized to separate molecular ions in the gas phase based on separation in time (time-of-flight mass analyzers, TOF), filtering (quadrupole analyzers), trapping (ion trap mass analyzers), as well as trapping with comprehensive signal processing (Fourier-transform ion cyclotron resonance (FT-ICR) and Orbitrap mass analyzers) [107, 108]. To facilitate deep analysis of complex biological samples, common proteomic approaches often utilize hybrid mass analyzers (quadrupole-TOF, ion trap-Orbitrap, etc.) and tandem mass spectrometry (MS/MS) approaches: separation of the molecular ions by their m/z in the first mass analyzer, fragmentation of ions with a variety of mechanisms, separation of the fragment ions by their m/z in another analyzer, and measurement of ion intensity of the molecular ions and their fragments [109]. The variety of peptide fragmentation mechanisms (collision-induced dissociation, CID; electron-transfer dissociation, ETD; electron capture dissociation, ECD; ultraviolet photodissociation, UVPD, and others) provide complimentary structural information on peptide sequences and post-translational modifications [110].

Commonly used analytical modes of MS include global proteome-wide approaches for protein identification (‘shotgun’ MS) and targeted approaches for protein quantification (parallel reaction monitoring, PRM; multiple reaction monitoring, MRM; and selected reaction monitoring, SRM, with the terms MRM and SRM often used interchangeably). Combinations of liquid chromatography (LC) separations with targeted MS measurements allow for the multiplexing of several hundred analytes and their measurements in dozens of biological and clinical samples [111–122]. MS has been an invaluable tool to identify the human proteome and reveal its complexity, post-translational modifications, and protein-protein interactions [123]. Targeted proteomics empowered with high-quality standards facilitated quantitative analysis of multiple proteins across disease and healthy states [124–132]. The high analytical specificity of MS facilitated measurements of monoclonal antibodies for their structural information, post-translational modifications, purity, and therapeutic levels in blood plasma [133]. Likewise, LC-MS has previously been used to quantify the levels of total IgG subclasses in serum [134]. However, the relatively slow progress of MS for serological diagnostics could be explained by insufficient MS sensitivity, the complexity of polyclonal antibodies (numerous isotypes, subclasses, allotypes, and variable regions), the lack of standards, and inability to predict sequences of hypervariable regions from the germline genomic sequences. While mass analyzers, analytical modes, sample preparation, and data analysis are continuously improving, conventional sandwich immunoassays are still 2–3 orders more sensitive than state-of-the-art LC-MS assays [135–137].

#### Mass spectrometry as a comprehensive detection technology for serological immunoassays

**Fig. 3:**
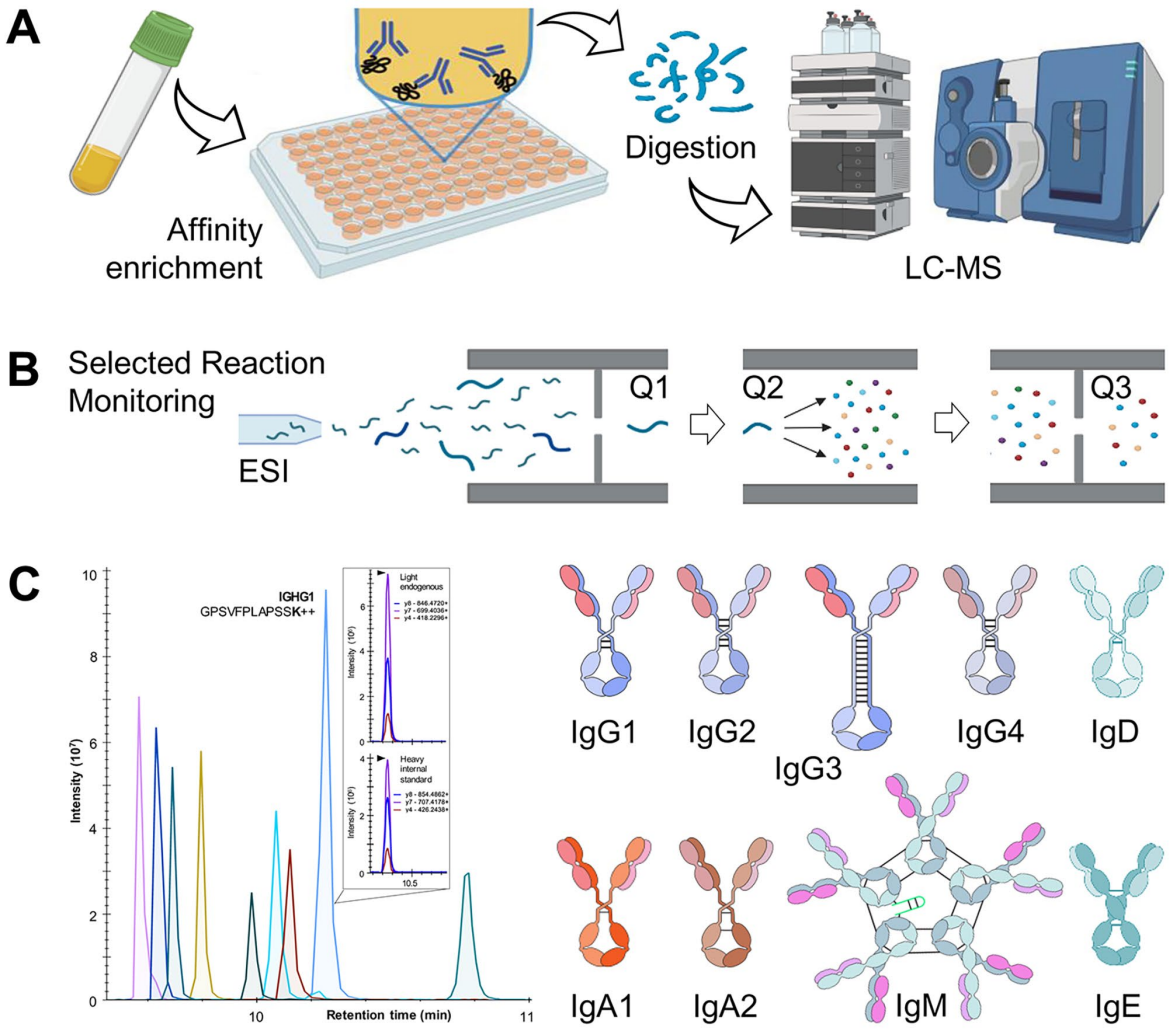
**Serological diagnostics with immunoaffinity-targeted proteomics**. **(A)** Specific antibodies are affinity enriched from the patient serum, denatured, and digested with trypsin, and the peptides are analyzed by targeted MS assays. **(B)** Following electrospray ionization (ESI), targeted MS assays, such as Selected Reaction Monitoring, enable the isolation of peptide ions of interest with the first quadrupole (Q1), their fragmentation in Q2, isolation of fragments of interest in Q3, and measurement of fragment ion intensities. **(C)** Peak areas of the “light” endogenous peptides and the corresponding spiked-in “heavy” isotope-labeled internal standards are used to calculate peptide ratios and absolute concentrations of the complete panel of the human immunoglobulin isotypes and subclasses

MS can be viewed as an emerging immunoassay detection technology, with the potential for proteome-wide identification and quantification of human proteins. Combinations of immunoaffinity enrichments with MS detection of proteins and peptides are known by a variety of terms including Mass Spectrometry Immunoassays [138], Stable Isotope Standards and Capture by Anti-Peptide Antibodies (SISCAPA) [139], Immuno-Precipitation Mass Spectrometry (IP-MS) [140], Immuno-MALDI [141], and others. Immunoaffinity-targeted proteomic assays (Fig. 3) provide a sensitivity of ~ 100 pg/mL in serum, approaching the sensitivity of common sandwich immunoassays (~ 10 pg/mL) [142, 143]. It should be emphasized that the readout of immunoassays with some conventional detection techniques (e.g. absorbance at 450 nm) is relatively simple and two-dimensional (wavelength and signal intensity). On the contrary, MS output is complex and multi-dimensional (LC retention time, precursor m/z, precursor intensity, fragment m/z, fragment intensity) and thus results in very high analytical specificity. Thus, the suggested IA-MS assays may well resolve the potential false positives and facilitate independent evaluation of diagnostic specificity of serological tests.

Recently, immunoaffinity proteomics was demonstrated as a conceptually novel platform for serological diagnostics [10, 144, 145]. Simple assay design and targeted proteomics measurements provided high sensitivity (1 ng/mL), high reproducibility (CV < 10%), and relatively high throughput (~ 100 samples/day) [10]. IA-SRM assays for testing new antigens can be rapidly developed and enable rapid response to epidemics and pandemics [10]. Advantages of IA-SRM assays in comparison to the conventional indirect immunoassays include (i) multiplex quantification of the complete panel of human immunoglobulin isotypes (IgG, IgM, IgA, IgE, IgD) and subclasses (IgG1-4, IgA1-2) in clinical samples; (ii) absolute quantification (ng/mL); (iii) high analytical specificity of MS which provided high diagnostic specificity (fewer false-positives); (iv) standardization using short synthetic stable isotope-labeled peptide reference standards which can be synthesized in large amounts and distributed across numerous clinical laboratories; (v) ~ 10-fold higher dynamic range (70–70,000 ng/mL for IgG1 in serum) which enabled measurements without multiple dilutions of clinical samples [10]. IA-SRM analysis of negative or positive convalescent COVID-19 plasma confirmed the true positive immune response with IgG1/IgG3/IgA1 pairing and provided a 385 ng/mL cut-off for anti-RBD IgG1 to detect COVID-19 convalescent plasma with nearly 100% sensitivity and specificity [10].

In the future, serological testing by IA-SRM may find its unique niche in clinical laboratories. There are ongoing initiatives to standardize protein measurements by mass spectrometry, such as the three-tier system using a fit-for-purpose approach for the discovery of protein biomarkers [146], and the Clinical and Laboratory Standards Institute (CLSI) C64 guideline “Quantitative Measurement of Proteins and Peptides by Mass Spectrometry” which provides a framework for the design, development, and validation of quantitative clinical protein and peptide assays [147]. While the throughput of IA-SRM is currently insufficient for large-scale population testing, IA-SRM may emerge as “gold standard” assays for independent validation and standardization of serological tests using stable, quantifiable, and affordable reference standards [10]. The relatively low throughput of IA-SRM assays could be improved through automation of IA and proteomic sample preparation, rapid digestion, fast microflow separations using short LC columns and sub-2 μm particles, multichannel turbulent flow LC, and scheduling for parallel analysis [10]. Rapid LC-independent approaches may include MALDI-TOF [148] and paper spray ion mobility MS [149] and may revolutionize serological diagnostics to the same extent as MALDI-TOF revolutionized clinical microbiology [150].

### Advancing immunoaffinity-proteomics for antibody sequencing

#### The challenge of antibody sequencing

Monoclonal antibodies are a rapidly growing class of innovative therapies with a recognized impact in oncology, autoimmune disorders, and chronic inflammation [151]. Sequencing of pathogen-specific antibodies enriched directly from patient samples may facilitate the rapid development of therapeutic antibodies. However, the immense diversity of antibody clones makes *de novo* sequencing a formidable challenge [152].

Variable heavy domains (VH) of human antibodies are assembled through the rearrangement of IGHV, IGHD, and IGHJ genes (V-D-J recombination) and subsequent affinity maturation, while the set of IGHC genes encodes the constant heavy (CH) domains and defines immunoglobulin isotypes and subclasses. Likewise, variable light domains (VL) are assembled through the rearrangement of IGKV, IGLV, IGKJ, and IGLJ genes (V-J recombination), while IGKC and IGLC genes define the constant light (CL) domains. VH and VL chains provide unique paratope conformations for the antigen binding with high affinity (K_d_ 0.01-10 nM), while the CH domains mediate protein-protein interactions with numerous Fc receptors and complement component 1 (C1) complexes to enable diverse immunological functions, such as complement-dependent cytotoxicity and antibody-dependent cellular cytotoxicity.

**Fig. 4:**
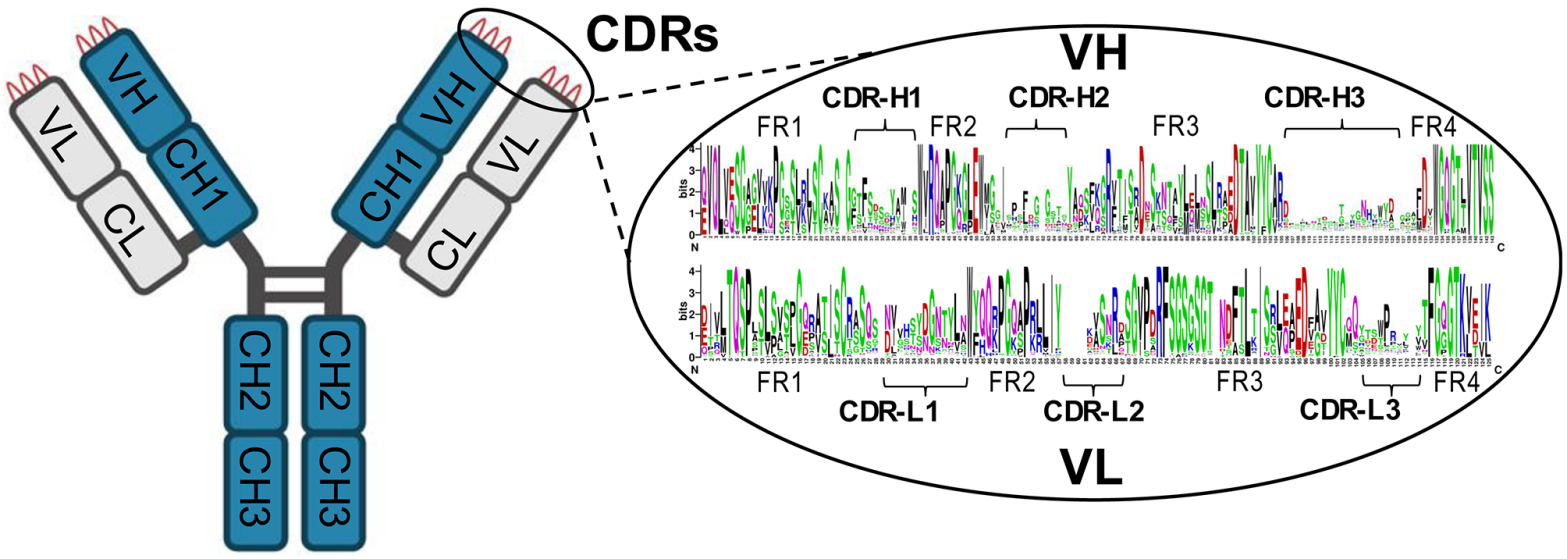
**Challenges of characterization and sequencing of polyclonal antibodies by MS**. Variable heavy (VH) and light (VL) chains of human immunoglobulins include semi-variable framework regions (FR1-4) and hypervariable CDRs, with CDR-H3 being the most diverse domain. Sequence logos present the experimental diversity of the matched heavy and light variable chains of 199 B-cell clones secreting anti-spike SARS-CoV-2 antibodies, based on reanalysis of data for a single convalescent donor [158]. The framework regions are encoded by the germline V- and J-genes, are minimally affected by affinity maturation, and include some well-conserved sequences (YYCAR, etc.) suitable as sequence tags for the hybrid *de novo* sequencing approaches

An antibody clonotype is defined as a group of sequences derived from a common progenitor B cell and sharing one of the variable (V), diversity (D), joining (J), and constant (C) genes [153, 154]. Recent studies on B cell receptor repertoire sequencing reported that the clonotype sharing between patients was much higher than expected by chance [155–158]. Inspection of immunoglobulin sequence diversity reveals semi-variable framework regions (FR1-4) and hypervariable CDRs, with CDR-H3 being the most diverse domain (Fig. 4). The framework regions are encoded by the germline V- and J-genes, are minimally affected by affinity maturation, and include some well-conserved sequences (YYCAR, etc.) suitable as sequence tags for the hybrid *de novo* sequencing approaches.

Numerous molecular biology, mass spectrometry, and bioinformatic approaches were developed to attempt antibody *de novo* sequencing [159]. MS sequencing of circulating antibodies was demonstrated using patient-specific proteomic databases generated through the single B cell receptor mRNA sequencing [159, 160]. Neural networks and deep learning approaches were developed to resolve complex and ambiguous antibody mass spectra and enable MS *de novo* sequencing without the patient-specific B cell receptor databases [161, 162].

#### Antibody sequencing with immunoaffinity-proteomics

IA-MS has the potential to advance *de novo* sequencing of high-affinity antibodies enriched from patient samples. Epitope-directed enrichments using short linear epitope antigens, increasing stringency (salts, pH, or temperature), and high-efficiency separation approaches may facilitate the enrichment of high-affinity, high-abundance, and low-diversity clonotype pools, as we previously demonstrated for aptamers and small molecules [163–169]. Our recent MS sequencing results for SARS-CoV-2 antibodies (unpublished), as well as other studies [157, 170], revealed that some pools of anti-RBD polyclonal antibodies included low numbers of clonotypes. While some of those high-abundance IGHV/IGKV clonotypes matched published B cell RNAseq data [171], additional high-abundance clonotypes potentially missed by the B cell RNAseq were identified. Intriguingly, even a single patient sample with a major and high-abundance antibody clonotype could be sufficient to sequence the most abundant clone, as demonstrated for a single patient with circulating sepsis-specific IgG1 [156]. Mass spectrometry sequencing of the mature high-affinity and high-abundance antibodies enriched directly from the patient’s samples will enable novel approaches to generate or refine therapeutic antibodies, extend the use of monoclonal antibody therapies, and advance precision immunology [172].

### Detecting T cell immunity with immunoaffinity-proteomics

#### Cell-mediated immunity

In addition to the humoral immunity mediated by B cell-derived antibodies, the adaptive immune system induces cellular immunity mediated by T cells. Briefly, CD8 + killer T cells or CD4 + helper T cells engage the specific T-cell receptors (TCRs) on their surface to search for human cells that display unique peptide antigens bound to the cell-surface Major Histocompatibility Complex (MHC) proteins. Recognition of a specific peptide-MHC complex by TCR triggers T cell activation and proliferation to facilitate either the direct cytotoxic activity and lysis of the infected cells (mediated by CD8 + cytotoxic T lymphocytes), or secretion of cytokines to induce proliferation of antibody-producing B cells (CD4 + helper T lymphocytes). The complexity of immune response emerges from the diversity of MHC proteins (three human MHC class I and three MHC II gene products with two alleles), diversity of T-cell receptor repertoires (VDJ recombination), and the numerous displayed peptides per each antigen (8–10 aa peptides for MHC Class I and 13–25 aa peptides for MHC Class II). Only the specific combination of peptide antigen, MHC allele and TCR results in pMHC-TCR interaction and T cell activation.

T-cell immunity has traditionally been evaluated with Enzyme-linked Immunospot (ELISpot) assays [173]. Such assays identify the specific peptide antigens that can activate patient-derived T cells. A sandwich immunoassay to detect interferon-γ secreted from activated T cells is used as a reporter system. While being very sensitive, ELISpot assays are tedious and low-throughput, require viable T cells, and reveal poor reproducibility across laboratories [174]. More robust and high throughput approaches to evaluate cellular immunity are urgently needed.

#### The prospects of T cell-mediated immunity evaluation with immunoaffinity proteomics

**Fig. 5:**
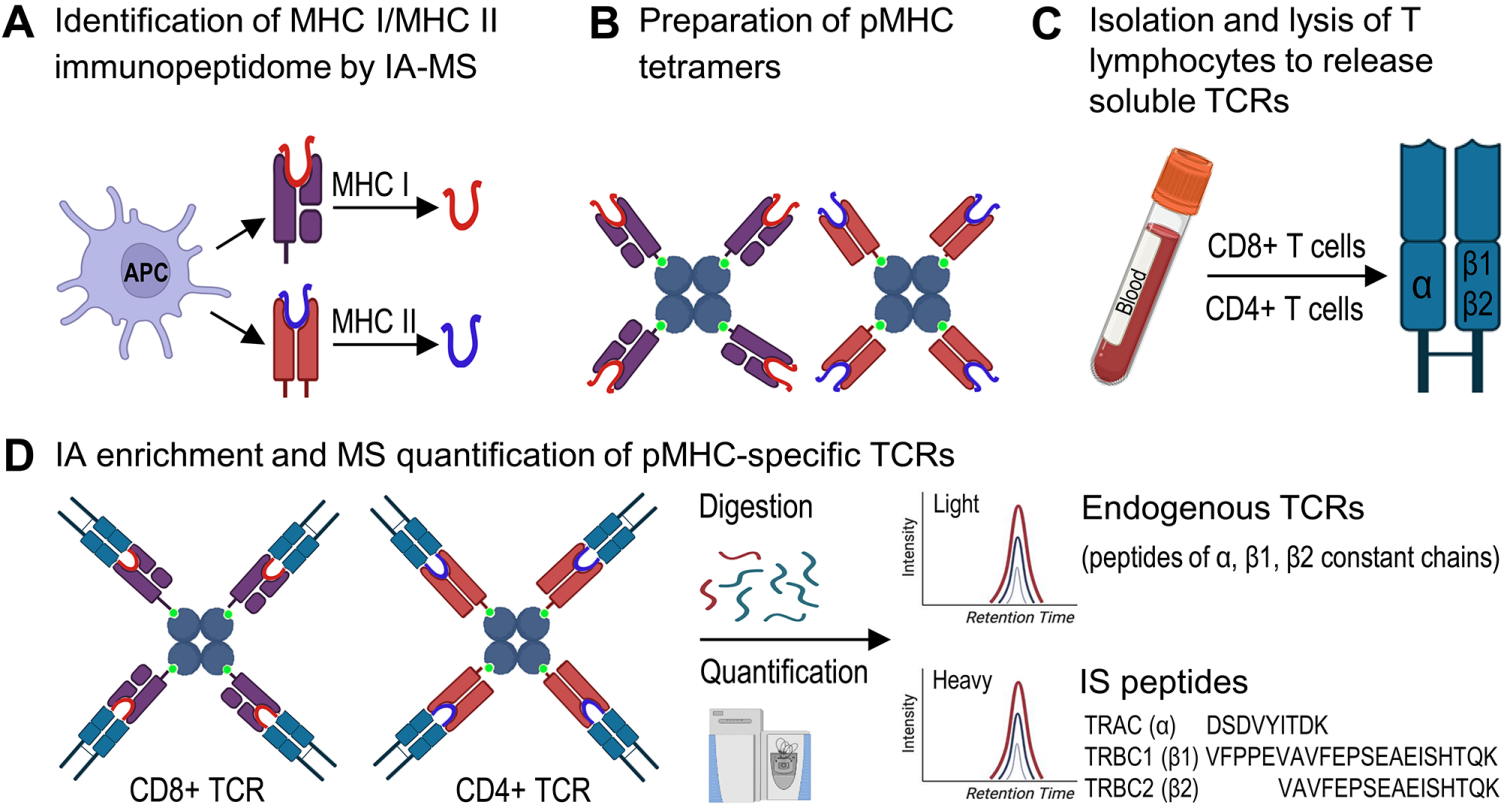
**The proposed IA-MS workflow for** in vitro **measurements of pMHC-specific TCRs**. (**A**) Immunopeptidome workflows facilitate the identification of MHC-specific peptides through the isolation of antigen-presenting cells, affinity enrichment of peptide-MHC (pMHC) complexes, dissociation of class I (9–10 aa) or class II (13–25 aa) MHC peptides, and *de novo* sequencing of MHC peptides by LC-MS. (**B**) Recombinant MHC I or MHC II proteins representing the patient-specific Human Leukocyte Antigen (HLA) variants are conjugated to magnetic particle-bound streptavidin tetramers and incubated with the synthetic MHC I or MHC II peptides previously identified with immunopeptidome workflows. Numerous pMHC complexes need to be prepared. (**C**) CD8 + cytotoxic T lymphocytes (MHC I) and CD4 + helper T lymphocytes (MHC II) are isolated from the patient’s blood and lysed to release soluble TCRs (α-β1 or α-β2). (**D**) T lymphocyte lysates are incubated with the individual pMHC complexes, and endogenous pMHC-specific TCRs are enriched and covalently cross-linked to pMHCs to retain low-affinity interactions. Following trypsin digestion, unique peptides of α constant (TRAC_HUMAN), β1 constant (TRBC1_HUMAN), and β2 constant (TRBC2_HUMAN) chains are quantified by LC-SRM. Corresponding heavy isotope labeled peptide internal standards (IS) facilitate accurate relative or absolute quantification

Recent proteomic approaches enabled the identification and *de novo* sequencing of MHC class I (8–10 aa) or class II (13–25 aa) peptides, epitope mapping, and identification of protein-protein interactions orchestrating the immune response [175]. Immunoaffinity proteomics may enable direct and in vitro evaluation of T cell-mediated immunity (Fig. 5). For instance, peptide-MHC complexes (pMHC) can be generated using the recombinant MHC I or MHC II proteins and matched synthetic MHC I or MHC II peptides, while the patient-specific soluble TCR pools can be generated with lysis of CD8+ (MHC I) or CD4+ (MHC II) T lymphocytes. Synthetic pMHC complexes will enable affinity enrichment of peptide-specific TCRs (with the optional chemical cross-linking to retain low-affinity interactions). The subsequent trypsin digestion and LC-SRM quantification of the unique peptides of α, β1 and β2 TCR constant chains will measure circulating levels of specific CD8 + or CD4 + T lymphocytes (Fig. 5).

The major challenges of evaluating T cell-mediated immunity with immunoaffinity proteomics will include (i) the immense diversity of HLA alleles (numerous polymorphic variants of the recombinant MHC monomers need to be multiplexed on a microplate); (ii) relatively low pMHC-TCR affinities (K_d_ ~1-100 µM [176]); (iii) inability to differentiate between viable and nonviable T cells. Development of proof-of-concept approaches for TCR identification, quantification, and sequencing may utilize some well-established immunotherapy constructs, such as affinity-matured TCRs binding with high affinity (K_d_~13 pM) to the recombinant pMHC complexes (such as melanoma-derived gp100 peptide displayed on HLA-A∗02:01) [177].

#### TCR sequencing

TCR sequencing, similar to BCR and antibody sequencing, presents a formidable challenge. The TCR repertoire is highly diverse and is generated through recombination, addition, and deletion of the various segments of the alpha and beta chains [178]. TCRs, however, do not undergo somatic hypermutation and affinity maturation. Interestingly, TCR diversity is greatly reduced in patients with some infections or myelomas [179]. In the future, highly multiplex immunoproteomic approaches may enable the identification of the dominant epitope-specific MHCs, assembly of the synthetic peptide-MHC complexes, and their use as baits to enrich specific TCRs for their *de novo* sequencing. TCR sequencing may revolutionize the development of efficient immunotherapies including CAR-T and autologous cellular immunotherapies [180].

## Conclusions

Serological assays were traditionally implemented as indirect immunoassays and their design has not changed for decades. The straightforward setup, speed of manufacturing, and affordability of indirect immunoassays were leveraged by their qualitative or semi-quantitative measurements, non-specific binding, cross-reactivity, lack of reference standards, and challenges with multiplexed measurements of isotypes and subclasses. Immunoaffinity proteomics provides an innovative platform for serological diagnostics to complement conventional indirect immunoassays and resolve their limitations. Clinical needs discussed in this review represent the areas for potential immediate improvement of serological testing. Even small improvements in diagnostic sensitivity and specificity of these assays may provide significant diagnostic benefits due to the large number of serological tests performed in clinical labs. The proposed immunoaffinity proteomic approaches may evolve into a routine serological testing platform and revolutionize serological diagnostics to the same extent as MALDI-TOF revolutionized clinical microbiology testing. Finally, novel approaches for fast and accurate *de novo* sequencing of human antibodies and TCRs isolated directly from patient samples may facilitate rapid and cost-effective development of high-affinity therapeutic antibodies and cellular immunotherapies, saving millions of lives worldwide.

## Data Availability

The datasets used and analyzed during the current study are available from the corresponding author on reasonable request.

## List of Abbreviations

ELISA: Enzyme-linked immunosorbent assay
IA-MS: Immunoaffinity-mass spectrometry
MHC: Major Histocompatibility Complex
SRM: Selected reaction monitoring
